# The Risk and Timing of Tuberculosis Diagnosed in Smear-Negative TB Suspects: A 12 Month Cohort Study in Harare, Zimbabwe

**DOI:** 10.1371/journal.pone.0011849

**Published:** 2010-07-28

**Authors:** Munyaradzi Dimairo, Peter MacPherson, Tsitsi Bandason, Abbas Zezai, Shungu S. Munyati, Anthony E. Butterworth, Stanley Mungofa, Simba Rusikaniko, Katherine Fielding, Peter R. Mason, Elizabeth L. Corbett

**Affiliations:** 1 Biomedical Research and Training Institute, Harare, Zimbabwe; 2 Wellcome Trust Tropical Centre, University of Liverpool, Liverpool, United Kingdom; 3 Clinical Research Unit, London School of Hygiene and Tropical Medicine, London, United Kingdom; 4 Harare City Health, Harare, Zimbabwe; 5 University of Zimbabwe College of Health Sciences, Harare, Zimbabwe; 6 Infectious Disease Epidemiology Unit, London School of Hygiene and Tropical Medicine, London, United Kingdom; MRC Laboratories, Gambia

## Abstract

**Background:**

Cases of smear-negative TB have increased dramatically in high prevalence HIV settings and pose considerable diagnostic and management challenges.

**Methods and Findings:**

Between February 2006 and July 2007, a cohort study nested within a cluster-randomised trial of community-based case finding strategies for TB in Harare, Zimbabwe was undertaken. Participants who had negative sputum smears and remained symptomatic of TB were follow-up for one year with standardised investigations including HIV testing, repeat sputum smears, TB culture and chest radiography. Defaulters were actively traced to the community. The objectives were to investigate the incidence and risk factors for TB. TB was diagnosed in 218 (18.2%) participants, of which 39.4% was bacteriologically confirmed. Most cases (84.2%) were diagnosed within 3 months, but TB incidence remained high thereafter (111.3 per 1000 person-years, 95% CI: 86.6 to 146.3). HIV prevalence was 63.3%, and HIV-infected individuals had a 3.5-fold higher risk of tuberculosis than HIV-negative individuals.

**Conclusion:**

We found that diagnosis of TB was insensitive and slow, even with early radiography and culture. Until more sensitive and rapid diagnostic tests become widely available, a much more proactive and integrated approach towards prompt initiation of ART, ideally from within TB clinics and without waiting for TB to be excluded, is needed to minimise the risk and consequences of diagnostic delay.

## Introduction

Southern Africa has experienced steeply rising TB incidence rates driven by the HIV epidemic, and now carries over three-quarters of the global burden of HIV-positive TB cases [1], [2]. In many high HIV prevalence countries the most striking increase has been in smear-negative TB cases [1].

In resource poor settings, smear-negative TB is difficult to diagnose and also difficult to exclude, especially in HIV-infected patients [3], [4]. Case series and post-mortem studies have shown the diagnosis of smear-negative TB to be highly specific [5]. However, the mounting evidence of low sensitivity of smear microscopy [6] and the high mortality, loss to follow-up and rate at which clinical deterioration occurs during diagnostic work up of HIV-positive smear negative TB suspects [7], [8] prompted a recent change in international management guidelines. In 2007, new guidelines [9] for use in settings with generalised HIV epidemics promoted diagnostic HIV testing of TB suspects, earlier use of radiography, increased access to sputum culture, a lower threshold for starting TB treatment in HIV-infected patients, and greater recognition of the symptoms of extra-pulmonary and disseminated TB (such as rapid weight loss and night sweats) in addition to chronic cough. For ambulant patients, a routine trial of broad-spectrum antibiotics in TB suspects was not recommended under these guidelines, but the need to exercise “clinical judgement” before starting TB treatment was retained.

We applied a standardised diagnostic protocol that predated but shared several key features of the new WHO guidelines, including early use of culture and chest radiography to a cohort of smear-negative TB suspects identified through a community-based intervention in Harare, Zimbabwe, who were then followed up for one year with active tracing of defaulters. The aims of the study were to investigate whether or not TB suspects had a high incidence of being diagnosed with TB in the year following initial work-up, suggesting missed diagnosis, and to investigate the role of routine culture in the diagnosis of smear-negative TB. Access to antiretroviral therapy (ART) was through the public health care system in Harare.

## Methods

This was a cohort study nested within a cluster randomised trial investigating two strategies (household enquiry vs. self-referral to a mobile community clinic) of community-based intensified case finding for TB in an urban community with a high prevalence of TB and HIV infection (DETECTB) [10].

### Ethical Approval

Ethical approval was obtained from the Medical Research Council of Zimbabwe, the Biomedical and Research and Training Institute, Harare and the London School of Hygiene and Tropical Medicine. Written informed consent was obtained from all participants. All participants were offered diagnostic HIV testing and counselling, with referral for assessment for ART if positive. Unreported HIV results taken for study purposes were held under a dedicated HIV test number, and only linked with other data through a computer programme that merged files and immediately dropped all personal identifiers, thus maintaining confidentiality.

### Study Setting and Population

Forty-six neighbourhood clusters in Western Harare, Zimbabwe, were provided with 6-monthly outreach access to TB microscopy at community-level as part of a cluster-randomised trial. The cohort reported here was drawn from participants in the cluster-randomised trial and who were recruited between February 2006 and June 2007. Inclusion criteria for the nested cohort study were: age 16 year or older; underwent outreach screening for TB in the community; had two negative sputum smears (morning and spot specimens); had ongoing symptoms of: cough; or weight-loss; night sweats; or a history of haemoptysis within the last year; and returned to take up our offer of follow-up investigations at the study clinic situated in a TB and HIV treatment facility.

### Initial Assessment for TB and HIV Testing

On first presentation at the TB and HIV clinic, initial assessment included recording of TB symptoms chest radiography, repeat morning and spot sputum smears and TB culture; and treatment with seven days of broad-spectrum antibiotics (amoxicillin). Participants with radiological features strongly suggestive of TB (including pleural or pericardial effusion) were referred for immediate TB treatment [9].

Additionally, on first assessment, all participants were asked to consent to provide an HIV specimen for study purposes and were advised to have diagnostic HIV testing and counselling (HTC), provided initially through routine testing and counselling services, and later through diagnostic HIV testing within the study clinic. HIV-positive participants were prescribed cotrimoxazole prophylaxis and referred to the adjacent public ART clinic where initiation criteria were CD4 count of ≤200 cells/ul, or a WHO stage 4 illness. From May 2006 all participants accepting diagnostic HTC also had blood taken for CD4 count. Participants who declined diagnostic HIV testing were asked for consent to provide a specimen for HIV testing for study purposes.

### Follow-up Procedures

Follow-up review for participants who were clinically stable was undertaken at the TB and HIV clinic 1, 8 and 12 months after initial assessment. Participants were advised to attend for unscheduled assessment and management if they became unwell. During follow-up assessments, participants underwent repeat assessment of symptoms and submitted further samples for sputum smear and TB culture. Chest x-ray was repeated if the participant was coughing. Participants with positive smears or cultures, with symptoms that did not respond to antibiotics, or with radiological signs of TB were referred for TB treatment. Participants who did not return for scheduled visits were followed-up at their home address.

Participants started on TB treatment were followed up at 1,8 and 12 months after commencing TB treatment. The 8 months review was chosen to allow assessment of TB treatment outcomes.

### Case Definitions for Diagnosis of TB

A confirmed TB case was defined as a positive smear (including scanty positive) or one or more positive cultures for *M. tuberculosis*. Smear- and culture-negative TB was defined by a clinical or radiological illness consistent with TB that did not respond to broad-spectrum antibiotics but did respond to one month of anti-tuberculosis treatment, or where TB treatment was started independently by an outside provider. Participants were classified as lost-to-follow-up if they did not attend for review (scheduled or unscheduled) during the study period and could not be traced.

### Laboratory Methods

Microbiology work was undertaken at the Biomedical and Research Training Institute, Harare, Zimbabwe. Smears were made from concentrated decontaminated (4% NaOH) specimens and read by fluorescence microscopy after staining with auramine-O. Positives were confirmed with Ziehl-Neelsen staining. Culture used Lowenstein-Jensen (LJ) slopes. Species were classified as *M.tuberculosis* or non-tuberculous mycobacteria according to colony morphology, microscopic cording, ability to grow on PNB-containing LJ media, and growth at 45°C, 37°C and room temperature. From 2008, MBP84 lateral flow assays for rapid species identification were also used.

Diagnostic HIV testing was offered with pre-and post test counselling (parallel testing with Determine™ HIV-1/2, Abbott Japan, Inverness Medical Japan, Tokyo, Japan and Uni-Gold Recombigen®, Trinity Biotech, Dublin, Ireland); unreported study HIV tests used serial testing (Abbott Determine™ with all positives and 10% of negatives confirmed by Uni-Gold Recombigen®) or oral mucosal transudate from participants not willing to provide serum, tested using Vironostika® Uni- form II (BioMérieux, Marcy l'Etoile, France).

### Statistical Analysis

We investigated TB free survival time from first clinical assessment to the earliest of last clinical review date, date of TB diagnosis or date of death. Incidence rates for outcomes were calculated using the Poisson distribution. Eight confirmed TB cases had missing data for date of TB diagnosis and so multiple imputation using chained equations, taking into account potential clustering, was used in these cases [11].

Baseline characteristics of participants were expressed as proportions and compared by HIV status using Fisher's exact test. We used Cox proportional hazard regression to assess the association of sex, age, weight, CD4 count and previous TB treatment with TB free survival, stratified by HIV status. Knowledge of HIV-status was not included due to high collinearity with unknown CD4 count category. Plots of Schoenfeld residuals were inspected to ensure the validity of the proportional hazards assumptions.

Data management and analysis was conducted using EpiInfo 2003 (CDC, Atlanta) and STATA 10.1 (College Station, Texas, USA).

## Results

### Baseline characteristics and follow-up

**Figure 1:**
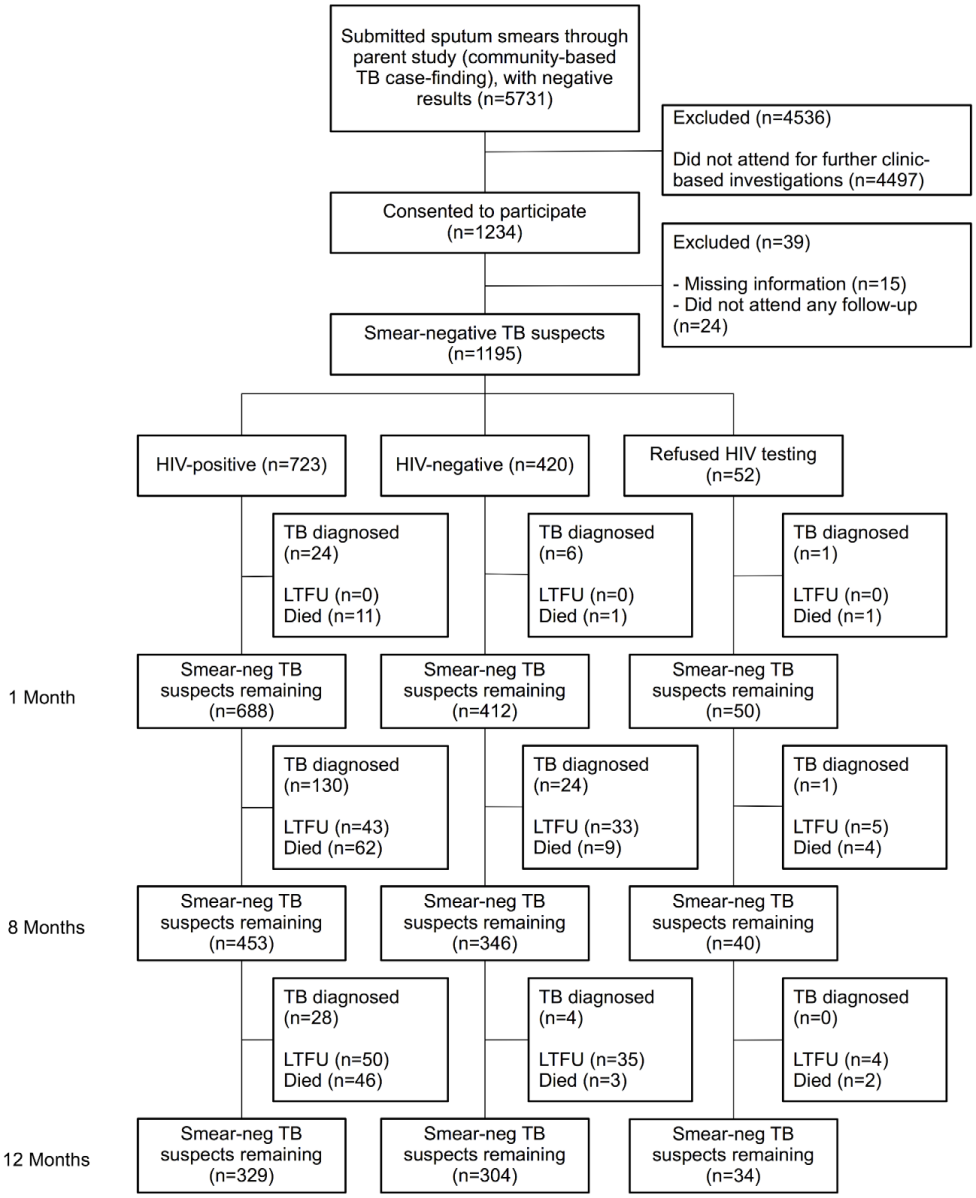
Cohort Flowchart. Diagnostic outcomes of smear negative TB suspects. LTFU: Lost to follow-up before TB diagnosis or in participant without TB diagnosed.

Between February 21, 2006 and June 6 2007, 5731 adults participated in the cluster-randomised parent study, and had two negative sputum smears. All participants in the parent study were provided with a clinic appointment card that could be used to obtain further investigations if smears were negative: 1,234 (21.5%) took up this opportunity and so form the starting cohort of this study (Figure 1). Thirty-nine (3.2%) participants were excluded from this analysis: 15 (1.2%) had missing baseline information and 24 (1.9%) did not attend any subsequent review after their initial appointment, leaving 1195 participants who were smear-negative TB suspects in this analysis (Figure 1). During the 12-month study period, 170 (14.2%) cohort participants were lost to follow-up. As reported separately, mortality rates were high with 97 participants dying without a diagnosis of TB and a further 42 participants dying after TB was diagnosed giving a total of 139 (11.6%) deaths during the study period (122.3 per 1000 person-years (95% CI: 104.4 to 144.3).

A total of 1143 participants underwent HIV testing with a further 52 (4.4%) refusing. Among participants who tested HIV-positive, 494 (68.3%) accepted diagnostic HIV testing and counselling (HTC) and 229 (31.7%) declined HTC but gave samples for unreported study testing. HIV prevalence was 63.3%. Baseline characteristics of HIV-positive and HIV-negative participants are shown in Table 1. HIV-positive participants were more likely than HIV-negative participants to be female (p = 0.019), and to have previously been treated for TB (p<0.001). They were also more highly symptomatic, with breathlessness at rest the only one of 12 considered symptoms that was not significantly more prevalent than in HIV-negative participants. Median CD4 count (limited to those accepting diagnostic HTC after May 2006: see Methods) was 149 (IQR 69 to 255) cells/ul: only 15.3% of counts were above 350 cells/ul, with a further 21.7% between 200 and 350 cells/ul.

**Table 1:** Baseline characteristics and symptoms of smear-negative TB suspects by HIV status.

Characteristic	Scoring	HIV+ (%)	HIV− (%)	P
**Total** *		**723 (63.3)**	**420 (36.8)**	
Sex	Female	470 (65.0)	243 (57.9)	0.019
	Male	253 (35.0)	177 (42.1)	
Age (years)	Median (IQR)	36 (30.0 to 42.0)	39 (30.0 to 54.0)	
	<25	60 (8.3)	47 (11.2)	
	25–34	261 (36.1)	111 (26.4)	<0.001
	35–45	264 (36.5)	95 (22.6)	
	>45	138 (19.1)	167 (39.8)	
CD4 count (cells/ul) (391 patients)	Median (IQR)	149 (69 to 255)	-	
	0–≤50	78 (19.9)	-	
	50–≤100	60 (15.3)	-	
	100–≤200	108 (27.6)	-	
	200–≤350	85 (21.7)	-	
	>350	60 (15.3)		
Previous TB treatment	Yes	138 (19.1)	35 (8.3)	<0.001
Chest x-ray: attending clinician classification	Normal	219 (30.8)	167 (39.8)	<0.001
	Typical of TB	27 (3.7)	7 (1.7)	
	Atypical abnormality, but consistent with TB	275 (38.0)	93 (22.1)	
	Suggests an alternative (non-TB) diagnosis	18 (2.5)	18 (4.3)	
	Not done	184 (25.5)	135 (32.1)	
**Symptoms**				
Cough greater than 3 weeks	Yes	543 (75.1)	263 (62.6)	<0.001
Sputum	Yes	401 (55.5)	168 (40.0)	<0.001
Purulent sputum	Yes	313 (43.3)	123 (29.3)	<0.001
Blood in sputum	Yes	145 (20.1)	37 (8.8)	<0.001
Duration with TB symptoms (weeks)	Median (IQR)	11.5 (4.0 to 20.0)	12 (4.0 to 24.0)	–
Chest pain	Yes	332 (45.9)	155 (36.9)	<0.001
Pleuritic painful cough	Yes	208 (28.8)	100 (23.8)	<0.001
Exertional dyspnoea	Yes	400 (55.3)	184 (43.8)	<0.001
Dyspnoea at rest	Yes	92 (12.7)	38 (9.0)	0.066
Night sweats	Yes	447 (61.8)	228 (54.3)	0.015
Change sheets or bedclothes due to sweats	Yes	326 (45.1)	127 (30.2)	<0.001
Recent weight loss	Yes	557 (77.0)	231 (55.0)	<0.001
Respiratory rate>20 breaths per minute	Yes	403 (55.7)	217 (51.6)	0.174

*HIV status not known for 52 (4.4%) participants.

IQR: Interquartile range, HIV+: HIV positive, HIV−: HIV negative.

### TB cases diagnosed during follow-up

From the 1195 participants, 218 (18.2%) TB cases were diagnosed in 1051.2 person-years of follow-up (Table 2). Eighty-six (39.4%) had confirmed TB and 127 (58.3%) were smear- and culture-negative. Five (2.3%) participants were treated for TB without clinical response, so failing to meet study case-definitions. HIV prevalence was 83.5% (95% CI: 77.8 to 88.2%) in participants diagnosed with TB.

**Table 2:** TB cases diagnosed during study period.

TB case definition	Total* (n = 218)	HIV+ (n = 182)	HIV− (n = 34)
	n (% of cases)	n (%)	n (%)
**Bacteriology confirmed** †	86 (39.4)	67 (36.8)	17 (50.0)
Time to diagnosis			
0 to 3 months:	60 (69.8)	51 (76.1)	8 (47.1)
>3 to 12 months:	26 (30.2)	16 (23.9)	9 (52.9)
**Smear- and culture-negative** ‡	127 (58.3)	110 (60.4)	17 (50.0)
Type of diagnosis			
Chest x-ray abnormal:	115 (90.6)	101 (91.8)	14 (82.4)
Clinical:	12 (9.4)	9 (8.2)	3(17.6)
Time to diagnosis:			
0 to 3 months	76 (59.8)	63 (57.3)	13 (76.5)
>3 to 12 months	51 (40.2)	47 (42.7)	4 (23.5)
**Case definition not met** §	5 (2.3)	5 (2.7)	0 (0.0)

*2 patients with TB declined HIV testing, both with smear- and culture-negative disease.

† 83 Pulmonary TB, 3 pleural TB.

‡ 105 Pulmonary TB, 9 pleural TB, 4 miliary, 6 other extra-pulmonary TB (ETB), 1 combined ETB and PTB, 2 not classified.

§ Treated for TB but case definitions were not met due to lack of response to treatment (4 pulmonary TB, 1 pleural TB).

HIV+: HIV positive, HIV−: HIV negative.

### Early (within 3 months) TB diagnoses

**Figure 2:**
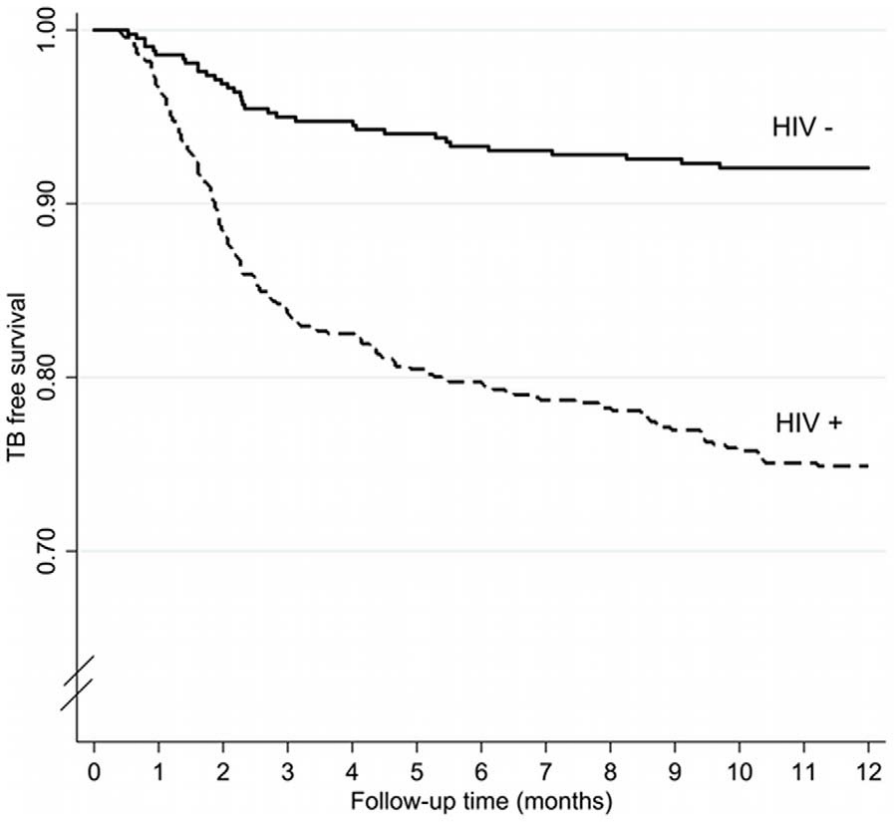
TB free survival stratified by HIV status. Kaplan-Meier estimates showing TB-free survival of cohort members stratified by HIV status.

TB free survival stratified by HIV status is shown in Figure 2. The majority of TB cases (139, 63.8%) were identified in the early period (0–3 months), and so are likely to represent prevalent disease at cohort entry. One hundred and seventeen (84.2%) of the participants diagnosed between 0 to 3 months were HIV-positive. HIV-stratified TB incidence within the first 3 months was 705.9 (95% CI: 580.8 to 860.2) and 205.0 (95% CI: 123.9 to 364.2) per 1000 person-years for HIV-positive and- negative participants respectively.

### Late (3 to 12 months) TB diagnoses

Between 3 and 12 months of follow-up, a further 79/218 (36.2%) TB cases were diagnosed. Incidence rates for this time period were 111.3 per 1000 person-years (95% CI: 86.6 to 146.3) in HIV-positive participants and 31.3 per 1000 person years (95% CI: 19.7 to 53.1) in HIV negative participants.

Bacteriologically confirmed disease was diagnosed more frequently amongst participants diagnosed with TB within the first 3 months (60/139; 43.2%) compared with participants diagnosed in the subsequent 9 months of follow-up (26/79; 32.9%; p = 0.151).

### HIV Positive Participants

51/117 (43.6%) had bacteriologically confirmed TB diagnosed within the first 3 months compared to only 16/65 (36.8%) in the subsequent 9 months (odds ratio (OR) = 2.4, 95% CI: 1.2 to 4.6; p = 0.016) in HIV positives individuals.

### HIV Negatives Participants

8/21 (38.1%) had bacteriologically confirmed TB diagnosed within the first 3 months compared to 9/13 (69.2%) in the subsequent 9 months (OR = 0.3, 95% CI: 0.1 to 1.2; p = 0.157) in HIV negatives participants.

### Delay in Diagnosis of TB

Median delay in diagnosis of bacteriologically confirmed and smear- and-culture negative disease TB was 2.0 (IQR: 1.2 to 3.4) months and 2.3 (1.5 to 5.4) months, respectively. HIV-negative participants had a longer delay in diagnosis of bacteriologically confirmed TB (3.1 months, IQR: 2.0 to 5.5) than HIV-positive (1.9 months, IQR: 1.1 to 3.0; p = 0.04), but similar delay to diagnosis of smear- and culture-negative TB (HIV-positive = 2.3 months IQR: 1.5 to 6.0; HIV-negative = 2.3 months IQR: 1.6 to 2.8; p = 0.61).

### Risk Factors for TB Diagnosis

HIV infection was the predominant risk factor for TB diagnosis, with TB incidence 3.5 times higher in HIV-positive participants (incidence 311.6 per 1000 person years, 95% CI: 266.7 to 364.9) compared to HIV-negative participants (incidence 81.8 per 1000 person-years, 95% CI: 58.3 to 118.2).

In univariable analysis among HIV-positive participants (Table 3), low weight (hazard ratio (HR): 2.11, 95% CI: 1.19 to 3.74) and CD4 count between 50 and 100 cells/ul (HR: 1.68, 95% CI: 1.08 to 2.62) were associated with an increased hazard of incident TB, while female sex showed a reduced hazard of TB (HR: 0.71, 95% CI: 0.50 to 1.01). These associations remained on multivariable analysis. On adjusted analysis there was an association between trend towards low CD4 count (p_t_ = 0.024), low weight (p_t_ = 0.026) and TB diagnosis. Among HIV-negative participants (Table 4), factors associated with a TB diagnosis in both univariable and multivariable analysis were a history of past TB treatment (adjusted hazard ratio (aHR): 2.18, 95% CI: 1.04 to 4.57), and older age (aHR: 2.07 [95% CI 0.88–4.89] for 35–45 years, 2.47 [95% CI: 1.22 to 5.02] for >45 years compared with <35 years).

**Table 3:** Univariable and multivariable analysis of risk factors associated with TB diagnosis in HIV-positive participants.

	TB cases/PYFU	Unadjusted HR (95% CI)	P-Value	Adjusted HR (95% CI)	P-Value
**Overall (0–12 months)**	182/584.1				
**Sex**					
Male	74/186.4	1.0		1.00	
Female	108/397.7	0.71 (0.50 to 1.01)	0.058	0.66 (0.47 to 0.94)	0.020
**Age (years)**					
<25	18/49.0	1.0		1.00	
25–34	68/213.6	0.85 (0.51 to 1.41)		0.94 (0.57 to 1.55)	
35–45	69/207.3	0.89 (0.54 to 1.47)		0.98 (0.56 to 1.71)	
>45	27/114.2	0.65 (0.32 to 1.32)	0.611	0.73 (0.32 to 1.64)	0.733
**Weight (kg)**					
0–49	47/98.4	2.11 (1.19 to 3.74)		2.05 (1.08 to 3.87)	
50–59	79/241.5	1.49 (0.83 to 2.66)		1.35 (0.73 to 2.52)	
60–69	40/168.6	1.13 (0.65 to 1.26)		1.08 (0.62 to 1.87)	
>70	16/75.6	1.00	0.008	1.00	0.026
**CD4 count (cells/ul)**					
<50	20/45.6	1.41 (0.82 to 2.43)		1.18 (0.69 to 2.01)	
50–<100	33/88.6	1.68 (1.08 to 2.62)		1.66 (1.04 to 2.64)	
100–<200	31/132.2	1.53 (0.90 to 2.59)		1.43 (0.82 to 2.48)	
≥200	77/259.6	1.0	0.091*	1.00	0.024*
Unknown	150/473.0	1.19 (0.80 to 1.79)		1.05 (0.68 to 1.62)	
**Previous TB treatment**					
No	150 (473.0)	1.0			
Yes	32 (111.1)	0.92 (0.59 to 1.42)	0.709		
**HIV status known** †					
No	51/214.5	1.0			
Yes	131/369.6	1.48 (1.04 to 2.12)	0.065		

*Test for Trend.

† Not included in multivariate model due to collinearity with CD4 count unknown category.

HR: hazard ratio; PYFU: person years follow-up; CI: confidence interval.

**Table 4:** Univariable and multivariable analysis of risk factors associated with TB diagnosis in HIV-negative participants.

	TB cases/PYFU	Unadjusted HR (95% CI)	P Value	Adjusted HR (95% CI)	P Value
**Overall (0–12 months)**	34/415.4				
**Sex**					
Male	16/169.3	1.00		1.00	
Female	18/246.0	0.79 (0.51 to 1.23)	0.301	0.82 (0.52 to 1.30)	0.403
**Age (years)**					
<25–34	7/159.6‡	1.00		1.00	
35–45	9/94.1	2.18 (0.92 to 5.13)		2.07 (0.88 to 4.89)	
>45	18/161.6	2.56 (1.25 to 5.24)	0.033	2.47 (1.22 to 5.02)	0.040
**Weight (kg)**					
0–59	19/167.5	2.45 (0.91 to 6.54)			
60–69	10/135.6	1.61 (0.53 to 4.89)			
>70	5/112.3	1.00	0.184		
**Previous TB treatment**					
No	28/381.3	1.00		1.00	
Yes	6/34.1	2.42 (1.12 to 5.24)	0.024	2.18 (1.04 to 4.57)	0.039

‡ Age groups <25 and 25–34 combined.

HR: hazard ratio; PYFU: person years follow-up; CI: confidence interval.

### Outcomes of TB Treatment

Two hundred and fourteen of 218 participants diagnosed with TB were started on TB treatment (4 were lost to follow-up in the interim) and followed for treatment outcomes. Ten participants defaulted from TB treatment and 10 were transferred out. Treatment completion rates were 69.6% (126/182) for HIV-positive and 75.0% (24/32) for HIV-negative participants, with case-fatality rates higher among HIV-positive (21.5%: 39 deaths on treatment) than HIV-negative (9.4%: 3 deaths, p<0.001) participants.

### Uptake of ART

Although 494 (68.3%) of the HIV-positive participants accepted diagnostic HTC and were referred for ART, only 106 (20.9% of those referred and 14.7% of all HIV positive participants) were started on ART during the 12-month follow-up. 145 (37.0%) of patients with known CD4 counts did not meet the ART initiation criteria (CD4 count of ≤200 cells/ul or WHO stage 4 illness), and limited availability of ART in the Zimbabwean public health care system was also a major constraint. Median time between TB diagnosis and ART initiation was 2.7 months (IQR: 1.1–7.1) and between study enrolment and ART initiation in those in whom TB was not diagnosed was 3.0 (IQR; 2.0 to 4.3) months.

## Discussion

The main finding from this large cohort of smear-negative TB suspects is the high incidence of TB diagnoses throughout 12 months of follow-up, for both HIV-positive and HIV-negative participants. HIV infection increased the risk of TB substantially in both early (within 3 months) and later periods.

In this cohort low weight and CD4 counts below 200 cells/ul were the main risk factors for subsequent diagnosis of HIV-related TB, while older age and past history of TB treatment were risk factors for HIV-negative TB. Past TB was a strong risk factor for recurrent disease in HIV-negative, but not HIV-positive, participants. This difference probably reflects the high susceptibility to rapid progression following re-infection with *M.tuberculosis* in HIV-positive individuals [12], [13].

TB was confirmed microbiologically in only 39.4% of TB cases, despite routine solid media culture for all TB suspects. With advanced HIV disease, sputum smears are more likely to be negative [14] and chest radiography may be normal [15]. Other studies from high HIV prevalence settings using combined clinical and bacteriological case-definitions and not restricted to smear-negative suspects have reported between 10% and 45% of patients as culture-negative [16]–[18]. The high rates of late diagnoses and low rates of culture-confirmed TB implies that diagnosing smear-negative TB remains problematic even with access to culture and early radiography [9].

We cannot distinguish between three explanations for the high TB incidence in the later period: a) that culture failed to detect true smear-negative TB initially, or b) misdiagnosis of other (non-TB) conditions as TB, or c) a high incidence of new TB during follow-up. But, given the consistent autopsy findings that disseminated TB is frequently missed in HIV-infected patients [19] and that response to TB treatment was documented in all but a few of our cases, we assume that delayed diagnosis of prevalent TB was the main contributor.

TB culture is the current international standard for diagnosis of smear-negative TB [20]. In low-prevalence HIV settings, TB culture on solid medium is highly sensitive (80–85%) and specific (98%) [21], although growth is slow (up to six weeks) and laboratory intensive [22]. In high prevalence HIV settings, sensitivity may be reduced [23], while the rapid pace at which HIV-related TB progresses makes waiting for culture results clinically inappropriate [7]. Liquid culture systems with early growth indicators provide more rapid and sensitive growth and could reduce diagnostic delay [3]. Other sensitive rapid diagnostics have shown great promise when evaluated against culture [24] but also need be evaluated against culture-negative disease, as the diagnostic barriers to management of TB suspects will not be completely resolved until rapid diagnostic methods with sensitivity approaching 100% become widely available [22].

By 12 months one quarter of our HIV-positive smear-negative TB suspects had been diagnosed with TB, highlighting the challenge that suspected HIV-related TB presents in the context of ART scale-up. Our finding that, rather than having disease excluded, participants continued to be diagnosed with TB at high incidence throughout follow-up supports a move towards starting ART promptly without allowing TB investigations to cause undue delay [25].

In this cohort, uptake of ART was extremely low and delayed despite low CD4 counts in most participants accepting diagnostic HIV testing. This in part reflects the unique limitations of public sector HIV care in Zimbabwe at the time, but other African ART programmes also report long delays between registration and ART initiation [26].

Based on these results, we suggest two revisions to the current recommended management of TB suspects. Firstly, lowering the threshold for TB treatment in HIV-infected TB suspects who do not have access to immediate ART. This would move away from the current recommendations of not starting TB treatment unless TB is considered likely towards prompt initiation of TB treatment within a few weeks of presentation as a TB suspect where there are still any clinical signs or symptoms consistent with TB. Inevitably this will mean more patients started on TB treatment, with some negative consequences: the specificity of the diagnosis of smear-negative TB would be reduced. More patients would also be subjected to the considerable costs and inconvenience of unnecessary TB treatment.

In many respects the preferable second alternative is prompt and early initiation of ART that would serve both a therapeutic purpose while also providing the means to rapidly confirm or exclude TB without leaving patients in the dangerous therapeutic limbo of having neither their confirmed HIV nor their possible TB treated. HIV is easy to diagnose, and ART “unmasks” TB [8]. Furthermore, a strategy of early ART initiation would ensure TB suspects are enrolled in structured follow-up where deterioration of symptoms can be promptly identified and managed. A recent randomised control trial has highlighted the benefit of early ART initiation for TB patients [27]. Our proposed strategy will increase the incidence of immune reconstitution inflammatory syndrome (IRIS) related to TB [28], especially in patients with advanced immunosupression [8], [29], but the risk of death from unmasking IRIS is low and likely to be considerably lower than the risk of death during prolonged clinical observation before ART initiation [30]. Although mortality may be lowered with this strategy, an increase in IRIS-related morbidity may be anticipated. This strategy could improve integration of TB and HIV services, and the CD4 count profile reported here suggests that ART initiation is warranted on CD4 count criteria in all but a small minority of HIV-infected TB suspects.

Uptake of ART by HIV-positive TB patients can be as low as 20% even in well-resourced ART programmes [31] unless careful attention is paid to avoiding the high costs incurred by multiple visits to different clinics [32]. This same phenomenon is likely to apply to TB suspects as well, whereby patients with limited resources are effectively forced to choose to invest either in continued TB investigations or in ART access [32], [33].

There were a number of limitations to this study. Firstly, as this cohort was drawn from participants in the parent cluster-randomised trial who self-presented for clinic assessment, there may be some differences from individuals in the general community and parent study who did not present for assessment. However, the large cohort number and the provision of a transport voucher to provide costs to access the clinic went some way towards mitigating this. As discussed, we cannot distinguish new incident disease from delayed diagnosis of prevalent disease. Clinic nurses undertaking assessment for TB were not blinded to participant's HIV status and this may have influenced their decision to commence TB treatment.

Over half of diagnoses were not bacteriologically confirmed, and so may have been incorrect despite documented treatment response. Our conclusions concerning culture may be less applicable to liquid than solid culture. Radiographs were read by attending clinicians, with no attempt beyond a weekly radiological meeting to monitor accuracy of interpretation, which is known to be highly subjective. Attempts were made to minimise bias in commencement of TB treatment by having explicit criteria for initial of TB treatment. Loss to follow-up was 14.2% in this cohort and it is possible that some of those who were lost to follow up in fact had TB, or died. In cohorts of HIV-positive individuals, mortality rates among those classified as lost to follow up are known to be as high as 50% [34]. A relatively low proportion of participants in this study were diagnosed clinically and this may reflect the use of TB cultures and chest radiology. Despite these limitations, our findings have important implications for the clinical management of smear-negative TB suspects in resource-limited settings.

### Conclusions

In conclusion, management of HIV-positive smear-negative TB suspects remains unsatisfactory when based upon early culture and radiology and routine HIV care services. Unless careful attention is paid to ensuring integrated and timely access to ART, patients may find themselves caught between programmes with signs and symptoms that are insufficient to prompt a clinical decision to start TB treatment, but are sufficient to delay acceptance into ART clinics. Integrated HIV and TB management to prioritise early initiation of ART, with all TB and HIV components ideally managed by the same clinic, may have more impact on the unsatisfactory scenario reported here and the current focus on improving diagnostic accuracy.
